# Supplementary material for: The interactome of intact mitochondria by cross-linking mass spectrometry provides evidence for coexisting respiratory supercomplexes
Source: Mol Cell Proteomics. 2017 Dec 8;17(2):216–32. doi: 10.1074/mcp.RA117.000470 (PMC5795388; doi:10.1074/mcp.RA117.000470)
Supplement: Supplemental Data [file supp_RA117.000470_133922_0_supp_23978_fzffwf.zip › spectra_annotation/mito_spectra_annotation/356-2-3-1-6-1.pdf]

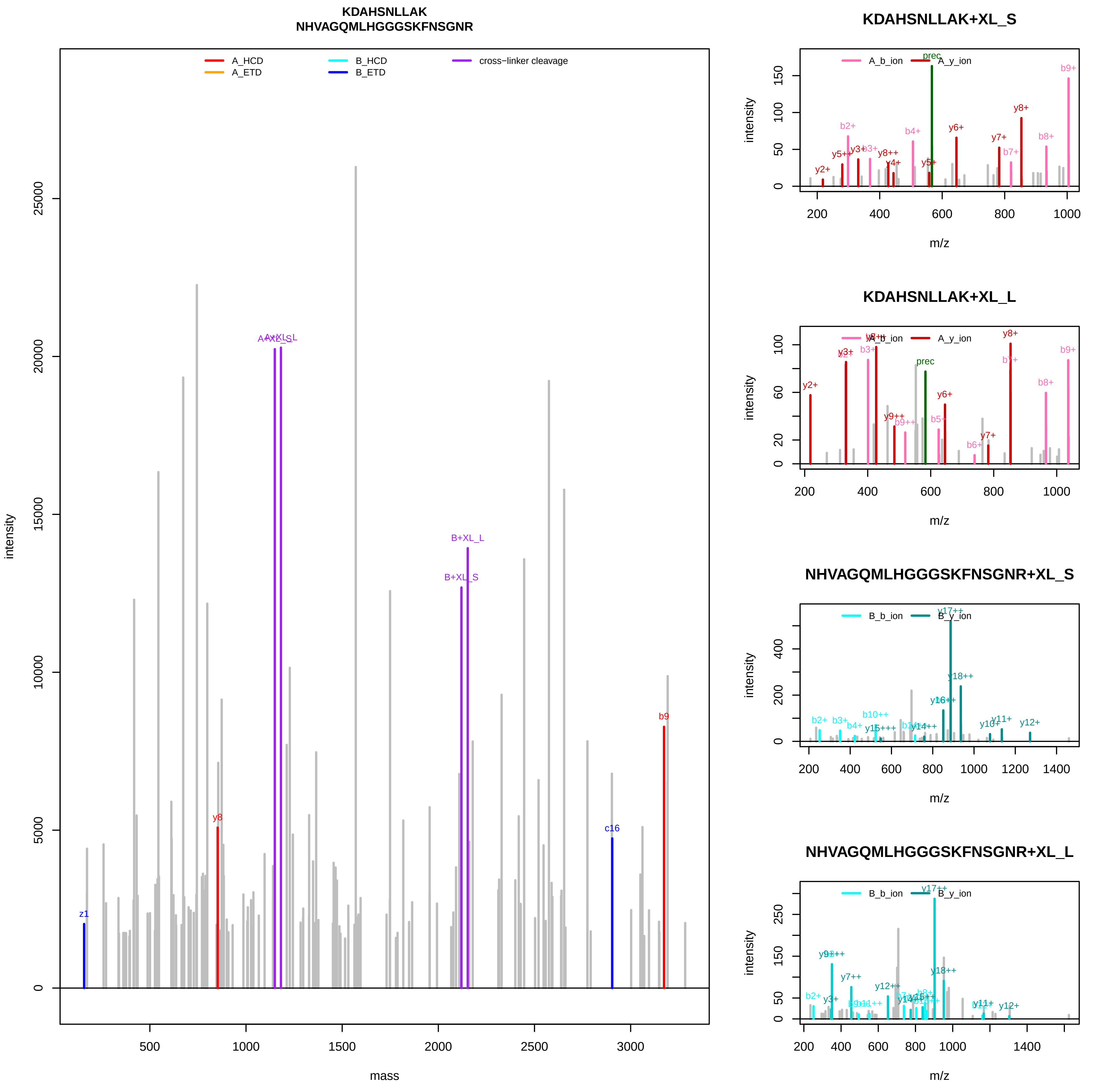
KDAHSNLLAK
NHVAGQMLHGGGSKFNSGNR
A_HCD
A_ETD
B_HCD
B_ETD
cross−linker cleavage
intensity
mass
z1
y8
A+XL_S
A+XL_L
B+XL_S
B+XL_L
c16
b9
KDAHSNLLAK+XL_S
A_b_ion
A_y_ion
prec
b9+
y8+
b2+
b4+
y6+
y7+
b8+
b7+
y2+
y5++
y3+
b3+
y8++
y4+
y5+
m/z
KDAHSNLLAK+XL_L
y2+
y3+
b3+
b7+
b8+
b9+
y6+
y9++
b9++
b5+
b6+
y7+
NHVAGQMLHGGGSKFNSGNR+XL_S
B_b_ion
B_y_ion
y17++
y18++
y16++
b10++
b2+
b3+
b4+
y15+++
y14++
y10+
y11+
y12+
NHVAGQMLHGGGSKFNSGNR+XL_L
y17++
y18++
y7++
y12++
b2+
y3+
y11+
y12+
